# Supplementary material for: Dynamics of collective action to conserve a large common-pool resource
Source: Sci Rep. 2021 Apr 28;11:9208. doi: 10.1038/s41598-021-87109-x (PMC8080682; doi:10.1038/s41598-021-87109-x)
Supplement: Supplementary file 1 — Supplementary Information. [file 41598_2021_87109_MOESM1_ESM.pdf]

Supplementary material: Dynamics of collective action to conserve a large common-pool resource

David Andersson*†, Sigrid Bratsberg†, Andrew K. Ringsmuth‡¶§||**, Astrid S. de Wijn †*
*Chemical Physics Division, Department of Physics, Stockholm University, Sweden
†Department of Mechanical and Industrial Engineering, Norwegian University of Science and Technology, Norway
‡Stockholm Resilience Centre, Stockholm University, Stockholm, Sweden
§Complexity Science Hub Vienna, Vienna, Austria
¶Section for the Science of Complex Systems, Medical University of Vienna, Vienna, Austria
||Current Address: Wegener Center for Climate and Global Change, University of Graz, Graz, Austria
**Current Address: Complexity Science Hub Vienna, Vienna, Austria

I. Parameter dependence

In order to ensure the fidelity of the model across the parameter space, we here present sweeps over the model parameters. In summary, when the parameters are varied, the model behaves as expected based on the findings presented in our main text. We comment below on the parameter dependence in the order of their appearance in figure 1. Here we used 200 network realizations per simulation.

A. External field (ϕ), fig 1a

Here we expand the interval over which the external field is varied. We see how an extremely strong positive external field (0.25) eliminates the initial decrease of the average state. This demonstrates that the external field is so strong that every single interaction increases the cooperativity. Beyond this point, increasing the external field further produces no qualitative changes. However, we see an even faster convergence to absolute cooperation.

At the other extreme, of low external field (0.01), we observe that the average state barely increases with time. The previous initial dip now resembles a drop in the average state, to a persistently low value. Decreasing the external field even further, we see a rapid drop to complete defection, from which the system never recovers. Its monotonic decline closely mirrors the increase we see for extremely positive external fields.

B. Stubbornness (w_i), fig 1b

Whereas one might expect a monotonic dependence of the cooperativity on the stubbornness, we instead see an optimal value at or near 0.6. We understand this intuitively as follows. At high stubbornness, the entire population is resistant to change and this slows the convergence. At low stubbornness, agents are extremely susceptible to the opinion of any agent they interact with and the convergence is dominated by the random selection of interaction partners, rather than by the dynamics of the interaction, resulting in a very small net increase.

Corresponding author: Astrid de Wijn (astrid.dewijn@ntnu.no)

C. Initial state, fig 1c

The total time to reach a certain proximity near the final state depends on the initial state, because for lower initial cooperativity, there are more defectors to convince. For unfavourable enough conditions the abundance of defectors will be so large that they completely overtake the cooperators, and the networks will on average go fully defecting. The red line for -0.5 levels off at a value not equal to 1 or -1. This is because we are plotting an average over many realisations of the network, and in this case the final result (1 or -1) is so sensitive to the initial conditions and realisation of the interaction that some networks become fully cooperative and some become fully defective. This is a finite-size effect.

D. Initial state standard deviation, fig 1d

We see that a larger variance in the initial state distribution corresponds to faster convergence. This can be understood from the grid figures in the main text. Some slightly cooperative agents must be present for cooperative clusters to form. The agent interaction (see Methods) is constructed such that agents on average can switch opinions only to other opinions that are presented to them. So, if no cooperators are initially present, none can appear subsequently either.

E. Friendship (w_{ij}), fig 1e

As friendship is implemented in the agent interaction almost identically to stubbornness, it is not surprising that the effects of changing the two are qualitatively similar. Friendship acts conversely to stubbornness: weak friendship makes it easier for an agent to retain their state and strong friendship makes the agent susceptible to changing its state.

F. Friendship standard deviation, fig 1f

Since we have chosen a favourable friendship value, giving it a large standard deviation introduces more nodes that either have very strong or weak friendships. This slows down the convergence, as described in the previous subsection.

G. Randomness, fig 1g

The randomness parameter in the interaction does not strongly influence the convergence rate. Each individual realisation can vary more when the randomness is large, but the average convergence does not change.

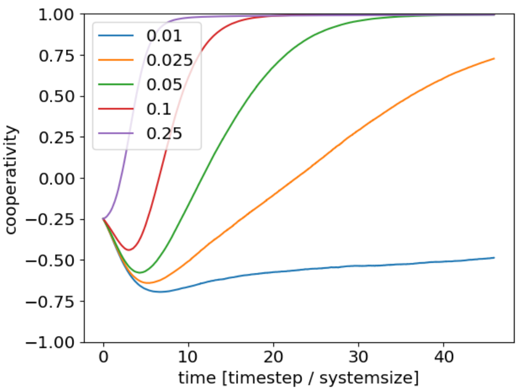

(a) External field

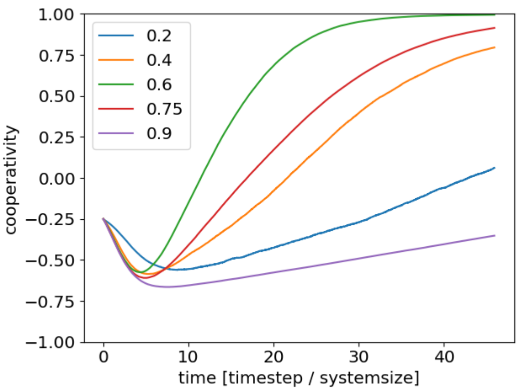

(b) Stubbornness.

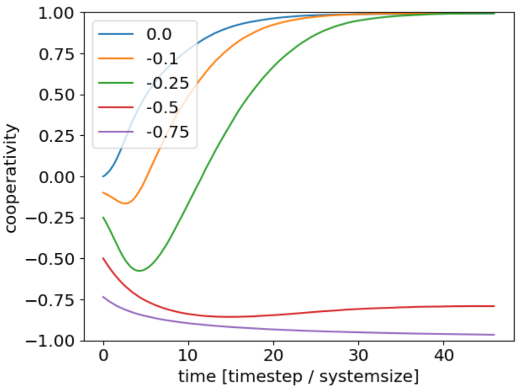

(c) Initial cooperativity.

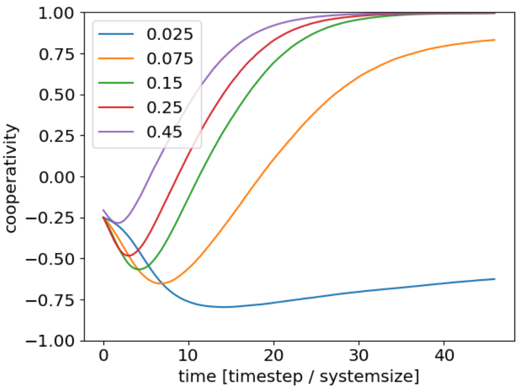

(d) Initial cooperativity standard deviation.

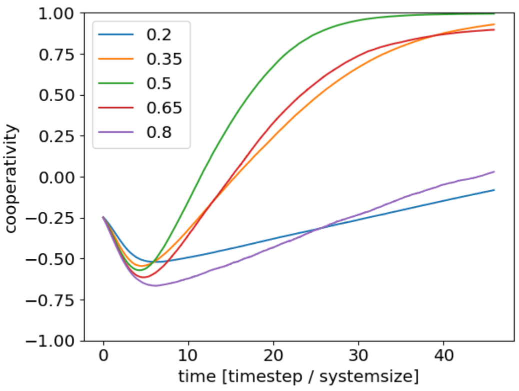

(e) Friendship.

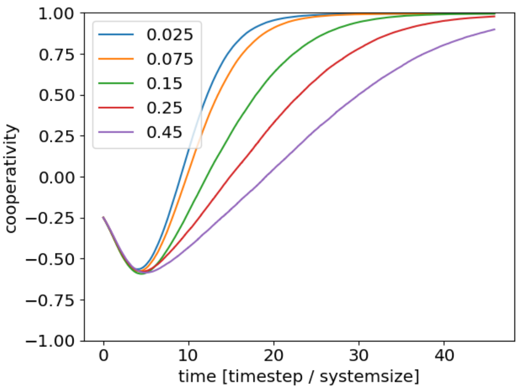

(f) Friendship standard deviation.

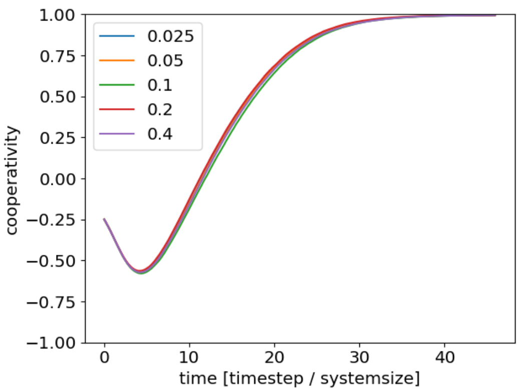

(g) Randomness.

Fig. 1: Summarizing parameter dependence of the model, c.f. table I in the methods section of the main text.
